# The Metabolomic Approach Reveals the Alteration in Human Serum and Cerebrospinal Fluid Composition in Parkinson’s Disease Patients

**DOI:** 10.3390/ph14090935

**Published:** 2021-09-17

**Authors:** Szymon Plewa, Karolina Poplawska-Domaszewicz, Jolanta Florczak-Wyspianska, Agnieszka Klupczynska-Gabryszak, Bartosz Sokol, Wojciech Miltyk, Roman Jankowski, Wojciech Kozubski, Zenon J. Kokot, Jan Matysiak

**Affiliations:** 1 Department of Inorganic and Analytical Chemistry, Poznan University of Medical Sciences, 60-780 Poznan, Poland; aklupczynska@ump.edu.pl (A.K.-G.); jmatysiak@ump.edu.pl (J.M.); 2 Department of Neurology, Poznan University of Medical Sciences, 60-355 Poznan, Poland; kpoplawska@ump.edu.pl (K.P.-D.); jolaflorczak@ump.edu.pl (J.F.-W.); wkozubski@ump.edu.pl (W.K.); 3 Department of Neurosurgery, Poznan University of Medical Sciences, 60-355 Poznan, Poland; bartoszsokol@ump.edu.pl (B.S.); klinkanch@op.pl (R.J.); 4 Department of Analysis and Bioanalysis of Medicines, Medical University of Bialystok, 15-089 Bialystok, Poland; wojciech.miltyk@umb.edu.pl; 5 Faculty of Health Sciences, Calisia University, 62-800 Kalisz, Poland; z.kokot@akademiakaliska.edu.pl

**Keywords:** Parkinson’s disease, cerebrospinal fluid (CSF), targeted metabolomics, lipidomics, liquid chromatography–tandem mass spectrometry (LC-MS/MS), amino acids

## Abstract

Parkinson’s disease (PD) is a major public health problem. Since currently there are no reliable diagnostic tools to reveal the early steps of PD, new methods should be developed, including those searching the variations in human metabolome. Alterations in human metabolites could help to establish an earlier and more accurate diagnosis. The presented research shows a targeted metabolomics study of both of the serum and CSF from PD patients, atypical parkinsonian disorders (APDs) patients, and the control. The use of the LC-MS/MS system enabled to quantitate 144 analytes in the serum and 51 in the CSF. This information about the concentration enabled for selection of the metabolites useful for differentiation between the studied group of patients, which should be further evaluated as candidates for markers of screening and differential diagnosis of PD and APDs. Among them, the four compounds observed to be altered in both the serum and CSF seem to be the most important: tyrosine, putrescine, trans-4-hydroxyproline, and total dimethylarginine. Furthermore, we indicated the metabolic pathways potentially related to neurodegeneration processes. Our studies present evidence that the proline metabolism might be related to neurodegeneration processes underlying PD and APDs. Further studies on the proposed metabolites and founded metabolic pathways may significantly contribute to understanding the molecular background of PD and improving the diagnostics and treatment in the future.

## 1. Introduction

Parkinson’s disease (PD) is a severe neurodegenerative disease, with a prevalence of 1% in the population older than 60 years [1]. When the degeneration of dopaminergic neurons in the substantia nigra reaches 60–80%, characteristic motor symptoms—including hypokinesia, postural instability, rigidity, and resting tremor—appear. To date, the diagnosis of PD is based on clinical symptoms [2]. It is difficult to diagnose PD in the early stage because premotor symptoms such as olfactory deficiency, constipation, sleep disorders, and depression are rather unspecific [3,4]. In the early stages of the disease, other neurodegenerative diseases, including multiple system atrophy (MSA), Lewy body dementia (LBD), progressive supranuclear palsy (PSP), and corticobasal degeneration (CBD), can mimic idiopathic PD [5]. These atypical parkinsonian disorders (APDs) have different prognoses and treatments, both in terms of standards and responses [3,4]. The therapeutic options in APDs are very restricted due to the lack of a clear etiology and because of overlapping clinicopathological correlations. Pharmacotherapy with levodopa (L-dopa) and other dopaminergic agents in APS have not been as successful as they are in Parkinson’s disease [6]. The golden standard for confirming PD remains neuropathological examination [7]. Because of the urgent need for earlier diagnostics, differentiating between PD and other parkinsonian syndromes, an emerging interest is to better understand the PD pathogenesis and metabolic changes due to disease [8]. Consequently, recent research has focused on the application of metabolomics to uncover early diagnostic and prognostic biomarkers in PD by the use of different analytical technologies. Preferably, these biomarkers should be accessible in non- or low-invasive samples such as blood, saliva, cerebrospinal fluid (CSF), or urine [9] and should reflect the underlying molecular mechanisms of the disease.

The pathogenesis of PD consist of a variety of mechanisms and multiple pathways such as alpha-synuclein [10], tau protein [11], amyloid beta misfolding [12], mitochondrial dysfunction [13], oxidative stress [14], calcium dyshomeostasis [15], axonal transport deficits [16], and neuroinflammation [17]. Previous studies have revealed the involvement of different pathways, such as glutathione [18,19,20], lipid [21], purine [3,22,23,24], energy [20,25,26], polyamine [27], tryptophane/kynurenine [19,20,23,24,28,29], fatty acid beta-oxidation [3,4,7,20,30], phenylalanine [28], and histidine [4] metabolisms. Trezzi et al. reported that early PD diagnosis could be based on increased levels of fructose, mannose, and threonic acid and decreased levels of dehydroascorbic acid in suspected PD patients [31]. 

Metabolomics is a relatively new technology that allows measuring the entire complement of metabolites, typically in a mass range of 50–1700 Da, in complex samples such as biological fluids or tissues [32]. In general, metabolomics studies can be targeted or non-targeted. Targeted analysis is restricted to a specific compound or a class of compounds, whereas non-targeted investigation provides a comprehensive chemical characterization of a complex bio/chemical system [33]. 

The presented targeted metabolomics approach allows to analyze both serum and CSF in the same way, with identical sample preparation procedures. Analyses were carried out using a high-performance liquid chromatography (HPLC) system coupled to a triple quadrupole tandem mass spectrometer with an electrospray ion source (LC/FIA-ESI-QqQ- MS/MS). A wide spectrum of compounds from different chemical classes was quantified, providing accurate information about the levels of many metabolites in both matrices. The aim of our study was to establish information about the levels of metabolites in human samples that allow the differentiation of PD from the control state or APDs and to gain insight into the molecular pathogenesis of the disease. 

## 2. Results

The targeted metabolomic approach was applied for quantitation of a wide spectrum of metabolites in the serum and CSF of patients of three groups: With PD, with APDs, and controls. The applied method enabled to quantitate up to 188 metabolites from such chemical compound groups as amino acids, biogenic amines, the sum of hexoses, acylcarnitines, glycerophospholipids, and sphingolipids. From those analytes, 144 were determined in the serum and 51 in the CSF, while the remaining analytes were excluded from further statistical analyses due to low abundance or presence only in part of the samples. For the study, well-established, validated, and robust methodology was applied [34]. The determined levels of metabolites were compared between groups of patients to assess their diagnostic utility in distinguishing challenging-to-diagnose cases as PD or APDs. Due to the urgent need for better diagnostic procedures for the early detection of neurodegenerative disorders, as well as better differential diagnosis, comprehensive and detailed basic research with appropriate statistical testing is of special interest. To find metabolites potentially useful for distinguishing patients with APDs and PD from healthy people, as well as for differentiating APDs patients from PD patients, ANOVA or Kruskal–Wallis tests were proposed. 

### 2.1. Serum Metabolome

The concentration of 18 out of 144 metabolites determined in the serum samples were statistically different between the compared groups based on one-way ANOVA or a Kruskal–Wallis test (Table 1). However, according to the post-hoc tests, only 14 metabolites were found as significantly altered between at least two from the analyzed groups, including one acylcarnitine, six amino acids/biogenic amines, and seven glycerophospholipids (Table 2).

**Figure 1:**
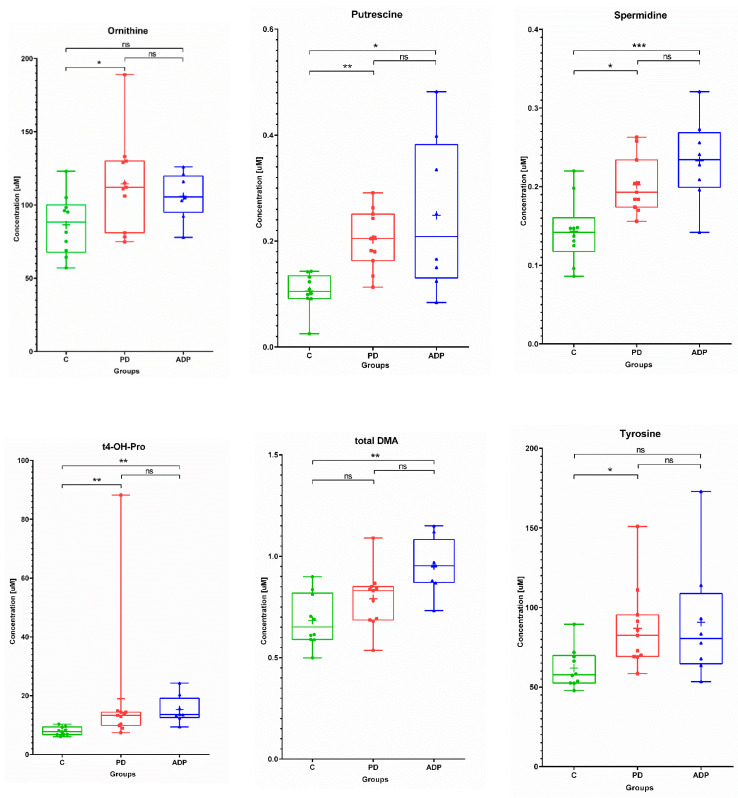
Box plots showing the distributions of the serum metabolites—amino acids and biogenic amines—found as altered between the studied groups: C—control group, PD—Parkinson’s disease, APDs—atypical parkinsonian disorders. Boxes extend from 25th to 75th percentiles; whiskers denote a range of measured concentrations; line and plus represent the median concentration and the mean of concentration in a group, respectively; dots, squares, and triangles show the measured concentrations in each group. * *p* ≤ 0.05, ** *p* ≤ 0.01, and *** *p* ≤ 0.001; ns—not significant.

None of the analyzed levels of sphingolipids were significantly different between the studied groups of patients. Considering the serum levels of amino acids and biogenic amines ornithine, putrescine, spermidine, trans-4-hydroxyproline, total dimethylarginine, and tyrosine distinguished at least two of the studied groups of patients. Among them, putrescine, spermidine, and trans-4-hydroxyproline were significantly different between the control and PD patients, as well as the control and APDs patients. The amino acids ornithine and tyrosine were statistically different between the control and PD patients, in contrast to total dimethylarginine, which was altered between the control and APDs patients. What is interesting is that a general trend of a lower concentration of those compounds in the control group was observed (Figure 1). This phenomenon might suggest that the changes in the amino acid profile are linked with processes related to neurodegeneration. 

**Figure 2:**
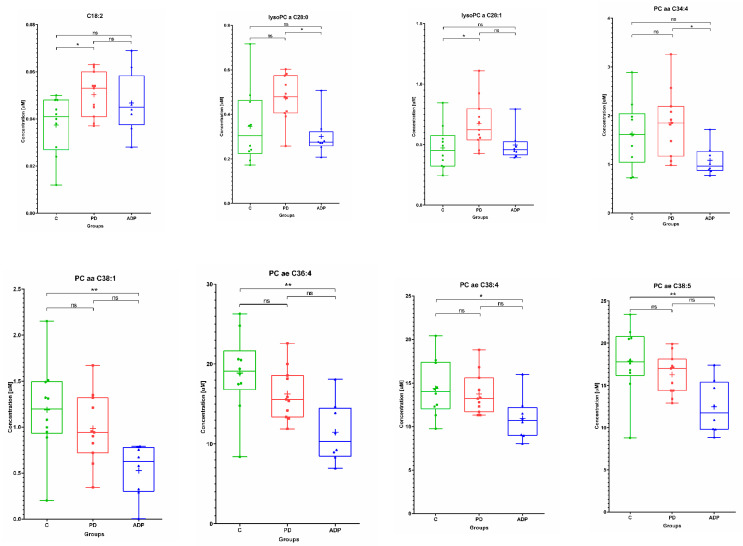
Box plots showing the distributions of the serum metabolites—in the class of lipids—found as altered between the studied groups: C—control group, PD—Parkinson’s disease, APDs—atypical parkinsonian disorders. Boxes extend from 25th to 75th percentiles; whiskers denote a range of measured concentrations; line and plus represent the median concentration and the mean of concentration in a group, respectively; dots, squares, and triangles show the measured concentrations in each group. * *p* ≤ 0.05, ** *p* ≤ 0.01; ns—not significant.

The lipid compounds showed a large scatter of results within each group (Figure 2). 

This is most likely related to a multitude of processes in which the lipid compounds are engaged. The single acylcarnitine found to be statistically significant in the presented study—octadecadienylcarnitine (C18:2)—was lower in the control group (control vs. PD). The opposite trend—higher concentrations of these lipids in the control group compared to APDs—was observed for glycerophospholipids: PC aaC38:1, PC ae C36:4, PC ae C38:4, and PC ae C 38:5. Generally, for these four lipids, the serum concentration was decreased in PD patients, as well as in APDs patients (Figure 2). Lysophosphatidylcholines selected as important at the means of PD: lysoPC a C28:0 and lysoPC a C28:1 were increased in the PD group. However, the level of lysoPC a C28:0 was significantly altered between the PD and APDs groups, in contrast to lysoPC a C28:1 (PD vs. control) (Figure 2).

### 2.2. CSF Metabolome

The analysis of the CSF metabolome showed that only four compounds were found as significantly altered between at least two from the analyzed groups: Tyrosine, putrescine, trans-4-hydroxyproline, and total dimethylarginine. 

In all comparisons, the levels of these metabolites in the CSF were lower in the control group (Table 3 and Table 4). Among the selected compounds, trans-4-hydroxyproline was significantly altered between the PD group vs. control, as well as the APDs group vs. control. 

**Figure 3:**
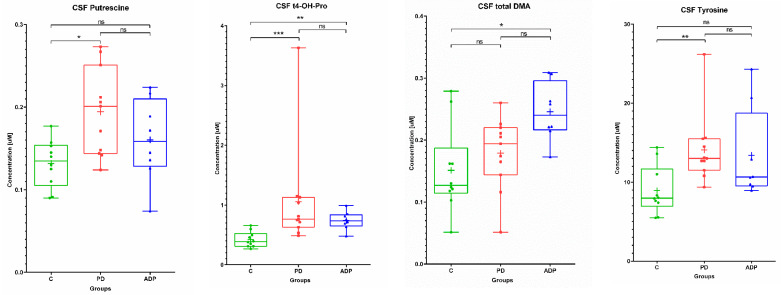
Box plots showing the distributions of the CSF metabolites altered between the studied groups: C—control group, PD—Parkinson’s disease, APDs—atypical parkinsonian disorders. Boxes extend from 25th to 75th percentiles; whiskers denote a range of measured concentrations; line and plus represent the median concentration and the mean of concentration in a group, respectively; dots, squares, and triangles show the measured concentrations in each group. * *p* ≤ 0.05, ** *p* ≤ 0.01, and *** *p* ≤ 0.001; ns—not significant.

What is worth highlighting is that the same trend was observed in the serum. Tyrosine and putrescine were found to be significantly different between the PD group and the control, in contrast to total dimethylarginine, which discriminated APDs from the control group (Figure 3). 

### 2.3. Pathways Analysis

**Figure 4:**
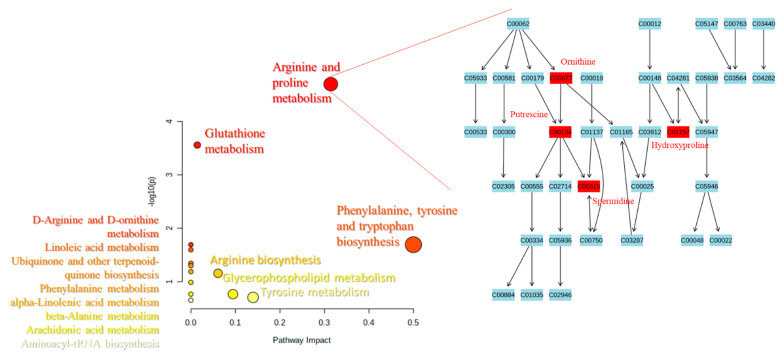
Metabolic pathways found to be potentially related to neurodegenerative processes, based on the identified potential biomarkers (left); the scheme of the arginine and proline metabolism pathway with significantly altered metabolites (red) between the studied groups of patients (right).

Based on the levels of compounds observed as altered between the studied groups of patients, we performed pathway analysis to select the metabolic pathways potentially correlated with neurodegenerative processes during the progress and development PD (Figure 4). The size and the position of the colored dots depict the importance of the analytes of interest on the appropriate pathway. The larger the dot, as well as the higher the coordinate values, the more important the pathway. The same might be supposed that the more involved a given metabolic pathway in the progress and development of the studied diseases. Figure 4 shows that arginine and proline metabolism might be an interesting target for revealing the biological background of PD and APDs and the underlying neurodegenerative processes. The results of the pathway analysis are in line with the previous observation—a significantly different level of trans-4-hydroxyproline in either the serum or the CSF in both comparisons: Control vs. PD group and control vs. APDs group. This finding might have a high impact in the future for improving diagnostic procedures. If confirmed in a greater number of patients, t4-OH-Pro seems to be a promising metabolite for patient screening. Other important pathways were also found to be potentially engaged in the studied disease, e.g., the glutathione metabolism pathway, which is in line with previous studies [18,19,20], as well as phenylalanine tyrosine and tryptophan biosynthesis (Figure 4.). 

## 3. Discussion

The amino acid proline has been an object of interest of researchers for years due to its involvement in a variety of functions: Apoptosis, autophagy, regulatory mechanisms, and signaling, not only in cancer studies, but also in aging and neurodegeneration [35,36]. We hypothesize that the proline pathway and its intermediates might be involved in the development of neurodegeneration, leading to, for example, PD. 

This study presented the results of the analysis of a wide profile of metabolites, applying the targeted metabolomic platform. The analytes included in this research, comprising amino acids, acylcarnitines, glycerophospholipids, sphingolipids, and the sum of hexoses, were measured in the serum and CSF of PD patients, APDs patients, and non-parkinsonian controls. We aimed to establish metabolic information that would allow for earlier diagnosis of PD or APDs and differentiation between PD and APDs. 

The concentration of ornithine—the immediate precursor of proline—was elevated in the serum of PD patients. An increased level of ornithine may cause hyperosmolarity in several brain regions such as the cerebellum, cerebral cortex, and brain stem by entering the urea cycle and increasing the urea concentration cycle [37]. The chronic osmotic pressure caused by ornithine damages plasticity in the hippocampal region by mediating tonic inhibition of the GABA receptor family. Furthermore, as a consequence of increased ornithine levels, proline levels rise and may thereby induce collagen biosynthesis and a shift of the immune system to a “wound-healing” program [37]. Similar results were obtained by Çelik et al., who showed an increased level of ornithine in the serum of Alzheimer’s and Parkinson’s patients [37]. 

Analyzing the CSF and the serum of PD and APDs patients, we found increased levels of trans-4-hydroxyproline compared to controls, but not between the PD and APDs groups. Jimenez et al. summed up the studies presenting blood amino acids evaluations, including some studies showing elevated levels of 4-hydroxyproline in PD patients [38]. Our results, indicating an increased level of hydroxyproline, are in line with these observations. Due to applying a targeted metabolomics approach, we observed in the serum of PD patients (vs. control group) decreased levels of proline but increased concentrations of hydroxyproline and glutamine. The same trends were observed in the CSF, but the differences were levels of proline that did not exceed the level of quantitation (data not shown). This finding allowed us to propose hypothetical interlink in compounds disturbed in PD. Since we found the significantly increased concentration of trams-4-hydroxyproline in PD, as well as in APDs, compared to control patients in both matrices (serum and CSF), the proline turnover should be elucidated more widely. We hypothesize that proline and hydroxyproline metabolism might play an important role in processes leading to neurodegeneration via a few potential mechanisms. 

These alterations might be directly related to neurodegeneration and disease progression. The most abundant reservoir of proline and hydroxyproline is collagen. These compounds constitute approximately 25% of all collagen amino acids. Hydroxyproline is formed by the hydroxylation of prolyl residues in newly synthesized collagen [39,40]. We suppose that the increased level of hydroxyproline in PD patients’ biofluids might be partially caused by the intensified degradation of collagen. The exacerbation of collagen degradation as a source of proline and hydroxyproline might be caused by matrix metalloproteinase (MMP) activity. These calcium-dependent zinc endopeptidases are responsible for the degradation and remodeling of tissues, e.g., by degradation of collagen. MMPs have been indicated as potential factors that contribute to the pathophysiology of PD [41]. 

**Figure 5:**
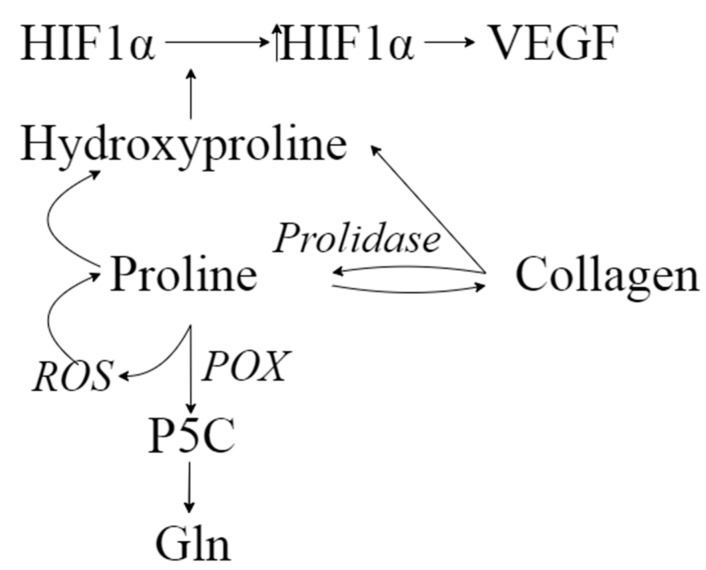
The hypothetical interlinks between the intermediates of proline–hydroxyproline conversion.

Neurodegenerative processes, including PD, might also be related to oxidative stress and mitochondrial dysfunction [42]. Transcription factor HIF-1α (hypoxia-inducible factor-1α) in hypoxic conditions triggers a variety of functions such as neuro- and cytoprotection. HIF-1α acts as a significant factor in the pathogenesis of PD. In physiological conditions, HIF-1α is degraded by prolyl hydroxylases; however, in the case of hypoxia, HIF-1α degradation is decreased due to lack of oxygen as a substrate, leading to an increasing level of HIF-1α [43]. An important observation was made by Surażyński et al. [44]: They examined the role of prolidase in the HIF-1 signaling pathway and found that hydroxyproline—the product of hydrolysis of collagen degradation products by prolidase—inhibits the degradation of HIF-1α. This effect was more potent for hydroxyproline compared to proline. Accumulated HIF-1α might contribute to enhanced angiogenesis through the increased expression of VEGF (Figure 5), and further to disturbances in vascularization, leading to neurodegenerative alterations. As reported previously, CSF biomarkers of angiogenesis are increased in PD, and they are associated with gait difficulties, blood–brain barrier dysfunction, white matter lesions, and cerebral microbleeds [45].

Proline is oxidized by proline oxidase (PRODH/POX) to pyrroline-5-carboxylic acid. This conversion leads to reactive oxygen species (ROS) formation, further triggering signaling cascades, including such processes as autophagy and apoptosis [46]. On the contrary, increased ROS formation might enhance the proline–hydroxyproline conversion. Liang et al. [47] indicated a possible direct reaction of proline with various ROS leading to the formation of, for example, 4-hydroxyproline (Figure 5). This evidence strongly suggests that proline turnover is crucial for body homeostasis. What is worth highlighting is that one of the crucial ways of utilizing proline is collagen biosynthesis [39]. It is especially interesting in light of a recent study by Raghunathan et al. [48]. They observed significant alterations between normal and PD-altered brains, such as elevated levels of extracellular matrix structural components, including collagens, among others. What is more, another study raised issues of the utility of hydroxyproline as an indicator of fibrosis [49]. Bearing in mind the aforementioned studies and the postulated alterations in proline metabolism, we hypothesize that there is a correlation between proline–hydroxyproline turnover and PD-related alterations in the structural components of the brain. 

In this context, our results regarding the alterations in proline and trans-4-hydroxyproline levels in PD patients seem to be especially interesting. The observed changes in the metabolite levels and PD molecular background are tough to directly interpret due to the complexity of proline metabolism. However, it sets a path for further multi-omics studies to describe the novel possible mechanism underlying neurodegeneration and to propose new therapies for the protection of dopaminergic neurons and new strategies for the development of new diagnostic markers.

Another potential mechanism of this phenomenon might be related to activity of DJ-1, a product of the *DJ-1/PARK7* gene. This multi-functional protein is responsible for antioxidation, regulation of transcription, and protein degradation [50]. A previous study proved the role of DJ-1 in the protection of brain cells from oxidative stress and correlated its mutation with autosomal recessive early-onset PD [51]. To play such various roles in the human body, this protein requires different partner proteins to interact with. The team of Yasuda [52] investigated pyrroline-5-carboxylate reductase 1 (PYCR1), the crucial enzyme involved in proline biosynthesis pathway, as a candidate for DJ-1 partner protein. They observed that DJ-1 binds to PYCR1 in vivo and in vitro, and they claimed that this protein might play a role in homeostasis between apoptotic proline and proapoptotic P5C through the balancing of PCYR1 activity in response to increased oxidative stress. Moreover, increased levels of the DJ-1 protein have been observed in the CSF of PD patients, suggesting the usefulness of its level as a biomarker for neurodegenerative disease [51]. Some therapeutic effects of DJ-1 and DJ-1-targeting molecules are also under investigation in animal models as potential therapeutic agents for neurodegenerative disorders, including PD [50]. Such a complex role of DJ-1 through both pro- and antiapoptotic action should be further investigated, even though the aforementioned studies show that the proline pathway with its intermediates seems to be unquestionably involved in processes related to neurodegeneration. 

Among the other findings of our research are the elevated levels of tyrosine in the CSF and serum of PD patients compared to the control group. Some studies measuring CSF amino acid levels have shown a significant increase in the tyrosine concentrations in PD patients compared to those of age- and sex-matched controls. Tyrosine and its metabolism seem to have a potential role in the etiology of neurodegenerative diseases [53,54]. The discussion on the role of tyrosine remains still open. Tyrosine is synthesized from phenylalanine in dopaminergic neurons of the brain. It is the precursor of L-dopa, which is converted into the neurotransmitter dopamine, which is, in turn, a precursor of noradrenaline and adrenaline. Increased tyrosine concentrations in the CSF of PD patients could hypothetically be related to an attempt by dopaminergic cells of the brain to increase the synthesis of dopamine in a situation of dopaminergic neuron depletion [38]. This statement is in line with our research on targeted metabolomics, where the levels of tyrosine in both of the studied matrices (CSF and serum) were elevated in PD patients compared to the control group.

We found higher serum concentrations of putrescine and spermidine in the PD and APDs groups compared to the controls. However, there was no statistical difference between the PD and APDs groups in the levels of these metabolites. Putrescine was elevated in the CSF of PD patients only. What is interesting is that polyamines have been isolated in α-synuclein depots and indicated to promote fibril aggregation in vitro [55]. It is possible that the subcellular co-localization of α-synuclein and polyamines in the cytoplasm makes their interaction possible [55]. Putrescine, spermidine, and also spermine accelerate the aggregation and fibrillization of α-synuclein, the major protein component of Lewy bodies and thus take part in the pathogenesis of PD [55]. Contrary to this theory, Büttner et al. [56] postulated that spermidine administration might have a cytoprotective function by induction of autophagy. Roede et al. reported significantly elevated N8-acetyl spermidine in the CSF of PD patients with rapid motor progression compared to both control subjects and slow progressors. The authors suggested that altered polyamine metabolism may be a predictive marker of rapidly progressing PD [27]. Moreover, an increased level of putrescine was noted in PD patients with incipient dementia (PDID) [57].

Increased levels of phosphatidylcholine, annotated as PC (34:4), and lysophosphatidylcholine (lysoPC C28:0) were found in the serum of PD patients compared to APDs patients. We found a higher serum concentration of lysophosphatidylcholine (LPC) C28:1 in the PD group vs. the control group. Contrary to our findings, decreased levels of PC 34:2 and 46:2, PC 34:5, 36:5, and 38:5, and total PC were observed in plasma and frontal cortex from PD patients [58,59] and in the substantia nigra from only male PD patients [60]. Stoessel et al. [61] showed alterations in several lipid classes, including increased levels of PC (44:6) and PC (44:5) and decreased levels of PC (35:6) in PD. They also showed the altered sphingolipid and amino acid metabolisms. Lysophosphatidylcholine (LPC) is the major class of lysophospholipids in the blood, and its levels are correlated with mitochondrial dysfunction, e.g., oxidation rate. Increased levels of LPC 16:0 and 18:1 have been shown in the lipid profiles of parkin-mutant fibroblasts compared to healthy controls [62]. An elevated concentration of plasma LPC 18:2 has been suggested as a biomarker for PD [63]. In an animal model of PD, LPC formation has been observed, which leads to cytotoxic changes, increased reactive oxygen species (ROS) formation in PC12 cells, reduced mitochondrial dysfunction, decreased dopamine release, and inhibition of its uptake [64]. Further studies are needed to understand changes in the levels of lipid species in PD. Studies should be focused on the CSF (and brain) lipidomes of PD patients, because plasma levels of lipids may not correlate with their brain levels. Moreover, many variables such as sex, age, PD etiology, microbiome, and DNA polymorphisms may have an impact on the lipid profile. In the current study, we found higher serum concentrations of octadecadienylcarnitine C18:2 in PD patients compared to the control group. Carnitine controls the mitochondrial energetic metabolism and peroxisomal oxidation of fatty acids [65]. Animal model studies have suggested that carnitine can reduce oxidative stress in aged animals [66] and is effective against the cell membranes damage caused by ROS [67]. Contrary to our results, Chang et al. showed a reduced level of octadecadienylcarnitine C18:2 in PD patients, which could be associated with mitochondrial dysfunction and ROS overproduction [68]. These mechanism might potentially be involved in the pathogenesis of PD [69]. 

Our study revealed an increased level of total dimethylarginine (tDMA) in the serum and CSF of APDs patients comparing to the control subjects. Asymmetric dimethylarginine (ADMA) inhibits nitric oxide (NO) synthase and diminishes endogenous NO levels. Increased serum ADMA levels cause a release of proinflammatory cytokines and are therefore related to oxidative stress and the inflammatory process [70]. Therefore, elevated ADMA level and diminished endogenous NO level indicate an increased risk of endothelial dysfunction, atherosclerosis, and numerous diseases such as atherosclerotic vascular diseases, hypertension, pre-eclampsia, dyslipidaemia, diabetes mellitus, kidney disease, asthma, and migraines [71]. Moreover, ADMA is considered to be a potential biomarker of subclinical vascular brain injury [72]. Arlt et al. [73] revealed that ADMA could be involved in AD pathogenesis. Because a reduced concentration of NO impairs the control of dopamine release and plays a role in emotional, behavioral, and cognitive deterioration, we speculate that higher serum and CSF concentration tDMA may play a role in the pathogenesis of atypical Parkinsonism. 

Each study has its own strengths and limitations. The limited numbers of samples in each group of patients is the main limitation of this work. In connection with this, it was impossible to perform within-group analyses of metabolites quantified in both the serum and CSF of PD patients depending on the severity of the disease. It should also be noticed that groups of patients should be homogenous for metabolomic studies. It is a tough task to select patients due to different lifestyle, pharmacotherapy, concomitant diseases, etc. However, appropriate exclusion criteria and strict medical interviews, as we proposed, might be helpful to include patients into studies, especially in further multicenter research. On the contrary, analysis of a wide spectrum of metabolites using the targeted metabolomic approach in two physiological fluids (serum and CSF) is one of the advantages. Moreover, by finding those metabolites whose concentrations differ significantly between compared groups of patients, as well as the proposition of specific pathways and mechanisms (e.g., proline metabolism pathway), we have set the path for further studies on a larger group of patients. Such a well-designed, prospective cohort studies might lead to a better understanding of the pathogenesis of PD and, furthermore, to improvement of its early detection.

## 4. Materials and Methods

### 4.1. Patient Recruitment and Diagnosis

All samples included in the study were collected in Heliodor Swiecicki Clinical Hospital in Poznan, Department of Neurology, Poznan University of Medical Sciences, Poland in accordance with the Declaration of Helsinki and its later amendments, as well as ethical standards of the institutional and national research committee. All participants accepted the goals of the research and signed informed consent prior to the study enrollment. The study protocol and the information for the patients were approved for the Local Bioethical Committee of Poznan University of Medical Sciences, Poland (decision nos. 821/16 of 2016 and 206/17 of 2017). The PD and APDs patients and control group were enrolled to the study based on physical examination, followed by a medical interview. 

The study included 29 subjects: 11 PD patients, eight patients with APDs, and 10 controls. All PD patients fulfilled the diagnostic criteria for PD according to the United Kingdom Parkinson’s Disease Society Brain Bank (UK PDSBB) criteria [74]. Five PD patients were in an advanced stage of the disease with motor complications from levodopa and levodopa-induced dyskinesias. The remaining six PD patients were in early stage of the disease with unilateral or mild bilateral involvement (stage 1 ± 2 on the modified Hoehn and Yahr rating scale, lack of motor complications from levodopa treatment, and a disease duration of less than four years). In the APDs group, four patients were diagnosed with MSA, three cases of MSA-P (multiple system atrophy with Parkinsonism), one case of MSA-C (cerebellar phenotype of MSA), two with PSP, and one person diagnosed with essential tremor and one with CBD. 

The exclusion criteria included alcohol and drug abuse, obesity, hypercholesterolaemia, liver damage, kidney failure, heart failure, autoimmunological disorders or other severe illnesses (e.g., infectious disease), and unusual diets (e.g., eliminating certain food groups). Patients after deep brain stimulation or receiving infusion therapy (levodopa-carbidopa intestinal gel or apomorphine pump) were also excluded, as well PD patients with dementia, while cognitive impairment in APDs was acceptable. 

All participants underwent a clinical assessment and provided serum and CSF samples in the course of clinical routine assessment and prospective studies. The concomitant diseases in the PD and APDs groups were hypertension, degenerative changes of the spine, and hypothyroidism (with thyroid and thyroid-stimulating hormones within normal limits). The L-dopa equivalent daily dose (LEDD) was calculated based on Clarke et al. [75]. All PD patients were treated with levodopa, 90% with dopamine agonists, 40% with amantadine, 30% with selegiline, and 20% with cholinolytics, as well as one patient receiving a neuroleptic—quetiapine. In the PD group, the most frequently used drugs for comorbidities were beta-blockers (20%) and statins (20%). Levothyroxine and acetylsalicylic acid were taken by 10% of patients. One patient (10%) also received dorzolamide with tymolol (10%). In the APDs group, seven patients were treated with levodopa, 25% with dopamine agonists, and 62.5% with amantadine. 

In the APDs group, the most frequently used drugs for comorbidities were valproic acid (25%, 2/8) and neuroleptic-quetiapine (25%, 2/8). One person was treated for hypertension. All controls lacked neurological or systemic diseases. The control patients underwent a lumbar puncture after presenting neurological symptoms (e.g., headache) to exclude diseases of the central nervous system. The general characteristics of the groups are described in Table 5.

### 4.2. Sample Collection

For this study, we obtained serum and CSF samples from a cohort of PD patients (*n* = 10) and controls (*n* = 10). The samples were collected after overnight fasting between 08:00 and 11:00 a.m. Peripheral blood was collected in S-Monovette R 2.7 mL, serum, 66 mm × 11 mm test tubes (Sarstedt AG & Co., Nümbrecht, Germany,) and centrifuged. CSF was collected and centrifuged for 10 min at 2000× *g* at room temperature. After centrifugation, the samples were aliquoted and stored at −80 °C in identical vials. 

### 4.3. Sample Preparation

For the targeted metabolomic assays, an AbsoluteIDQ^®^ p180 Kit (Biocrates Life Sciences AG, Innsbruck, Austria) was used. The proposed methodology enabled analysis of up to 188 metabolites, comprising amino acids, biogenic amines, acylcarnitines, glycerophospholipids, sphingolipids, and hexoses in both the serum and cerebrospinal fluid. 

The blinded samples were analyzed in a random order, taking into account the type of material analyzed. As is widely known, metabolite levels can be significantly lower in CSF compared to human serum or plasma; thus, different amounts of the biological matrix were put onto a kit plate: 10 µL and 30 µL of serum and CSF, respectively. All of the procedures related to sample preparation and metabolite quantitation were in accordance with the manufacturer’s recommendations. 

In the first step, basic reagents, mobile phases, and wash solvent were prepared. Sample preparation started from sample registration and their randomization, which enabled to generate a sample list with a plate layout with localization of blank, QC samples, calibrators, and individual samples on a 96-deep-well plate. Directly prior the assay, the human samples were thawed, vortexed, and centrifuged for 5 min at 2750× *g*. Internal standards’ mixture, prepared in advance, was spotted directly into the wells, omitting the spot of a blank sample. After pipetting the appropriate solutions of prepared phosphate buffered saline for the blank samples, QC samples, calibration standards, and the patients’ samples were pipetted on the spots according to the plate layout. Then, the plate was placed in a nitrogen-positive pressure manifold for 30 min for drying the spots under nitrogen flow. At the time of drying, the phenylisothiocyanate (PITC) derivatization solution was prepared and then pipetted into each well in the amount of 50 µL. After this step, the plate was covered for 20 min, enabling taking place the reaction. Then, the lid was removed and the plate was dried in the nitrogen flow for 1 h. In the next step, 300 µL of the extraction solvent was added to each well, and the plate was shaken for 30 min at 450 rpm using an orbital shaker. Furthermore, positive pressure manifold was used to pass the extracts through the filter layer to the capture plate. Finally, the extract was split and appropriately diluted for performing a liquid chromatography–tandem mass spectrometry (LC-MS/MS) experiment and a flow injection analysis–tandem mass spectrometry (FIA-MS/MS) experiment. The implementation of the method was described by us earlier [76].

### 4.4. Instrumentation and Metabolite Assays

A high-performance liquid chromatograph 1260 Infinity (Agilent Technologies, Santa Clara, CA, USA) coupled to a 4000 QTRAP (SCIEX, Framingham, MA, USA) mass spectrometer with an electrospray (ESI) ion source was used. Analyses of amino acids and biogenic amines were preceded by chromatographic separation by means of gradient elution, at the constant flow rate of 0.5 mL/min with ZORBAX Eclipse XDB-C18 (3.0 mm × 100 mm, 3.5 µm) column (Agilent Technologies, Santa Clara, CA, USA), with a pre-column (C18, 4.0 mm × 3.0 mm) SecurityGuard (Phenomenex, Torrance, CA, USA). The remaining groups of metabolites were analyzed in FIA mode. The parameters of the LC-MS/MS and FIA-MS/MS methods were set according to the manufacturers’ instructions.

System suitability tests (SSTs) and blank samples were analyzed to verify the overall performance and system purity. For compound detection, multiple reaction monitoring mode in positive and negative ionization mode was applied. Data management and acquisition were carried out under the control of the Analyst (SCIEX, Framingham, MA, USA) and MetIDQ (Biocrates Life Sciences AG, Innsbruck, Austria) software. The reliability of the method was proved by an interlaboratory reproducibility test, among others [34].

### 4.5. Data Analysis

Quantitative data are presented as micromoles. To determine the most relevant differences in the metabolite concentrations between the compared groups of patients, the following statistical tests were conducted. First, the normality of the distribution was tested based on a Shapiro–Wilk test. Next, for normally distributed variables, the equality of variances was confirmed by Levene’s test. For a normally distributed set of variables with confirmed equal variances, one-way analysis of variance (ANOVA) was applied. The remaining metabolites, i.e., with non-normally data distribution or unequal variance metabolites, a Kruskal–Wallis test was conducted. To discover which of the study groups differed significantly in terms of the level of individual metabolites, post-hoc tests were performed. For one-way ANOVA, the Tukey honestly significant difference (HSD) test for multiple comparisons was performed. Similarly, for the variables chosen by the Kruskal–Wallis test, post-hoc analyses were carried out. The performed statistical analyses were conducted using STATISTICA software version 13 (StatSoft, Tulsa, OK, USA). For the statistical data visualization, GraphPad Prism (GraphPad Software, San Diego, CA, USA) was used. 

The metabolites found to be altered between at least two from analyzed groups were matched to HMDB id and included in the pathway analysis using MetaboAnalyst 5.0 web server [77]. The pathway analysis parameters were as follows: Visualization method—scatter plot (testing significant features); enrichment method—hypergeometric test; topology analysis—relative-betweenness centrality; using the Homo sapiens (KEGG) pathway library. MetaboAnalyst 5.0 web server and draw.io—free online diagram software were used for the pathway analysis visualization. 

## 5. Conclusions

To summarize, in the current research, we presented a wide-spectrum targeted metabolomic profiling of different classes of metabolites in serum and CSF collected from patients with PD, APDs, and the control group. We demonstrated increased serum levels of phosphatidylcholine (PC 34:4) and lysophosphatidylcholine (lysoPC C28:0), potentially differentiating PD from APDs. This differentiation (PD vs. APDs) is, for now, very challenging and time-consuming and is very often based only on the clinician’s experience. Thus, our findings shed light on the possibility of using metabolomic and lipidomic profiling for better, earlier, and faster diagnosis of PD and APDs. Elevated levels of tDMA in the serum and CSF differentiated APDs patients from the control group. Higher serum and CSF concentrations of tyrosine, putrescine, and trans-4-hydroxyproline differentiated PD patients from control subjects, but no significant differences were observed between PD and APDs patients. However, it is still strongly suggested that trans-4-hydroxyproline is engaged in processes related to neurodegeneration. Our studies present evidence that the proline metabolism pathway might be related to neurodegeneration processes underlying PD and APDs. To the best of our knowledge, for the first time, we proposed hypothetical interlinks between intermediates of proline–hydroxyproline conversion in PD patients. Moreover, we observed increased serum levels of octadecadienylcarnitine C18:2, ornithine, and spermidine, which differentiated PD patients from the control group but not PD patients from APDs patients. The main strength of our study is an analysis of two physiological fluids (serum and CSF), whereas the main limitation is related to the limited numbers of samples. In future research, the group of patients should be divided depending on the severity of the disease and on their pathophysiology, i.e., tauopathies and synucleinopathies. Well-designed prospective studies on a larger group of patients are essential for the search for a novel biomarker and for a better understanding of the pathogenesis of PD and atypical Parkinsonism.

## Figures and Tables

**Table 1:** A list of the metabolites determined in the serum of patients and identified as significantly different between the analyzed groups based on either analysis of variance (ANOVA) or non-parametric Kruskal–Wallis ANOVA, depending on the normality of distribution and the variance equality. All values are expressed in micromoles.

Serum										
No.	Metabolite	Control (*n* = 10)			PD (*n* = 11)			APDs (*n* = 8)		
		Mean	SD	Median	Mean	SD	Median	Mean	SD	Median
1	C18:2	0.04	0.01	0.04	0.05	0.01	0.05	0.05	0.01	0.05
2	Ornithine	86.43	20.57	88.30	114.47	32.3	112.00	105.91	15.63	105.50
3	Tyrosine	61.88	12.51	57.80	86.95	25.96	82.5	90.75	38.16	80.55
4	Putrescine	0.11	0.03	0.11	0.20	0.06	0.21	0.25	0.14	0.21
5	Spermidine	0.14	0.04	0.14	0.20	0.04	0.19	0.23	0.05	0.23
6	t4-OH-Proline	7.96	1.41	7.80	18.95	23.11	13.30	15.29	4.78	13.55
7	Total DMA	0.68	0.13	0.65	0.79	0.14	0.83	0.95	0.14	0.95
8	lysoPC a C26:0	0.31	0.14	0.28	0.48	0.19	0.38	0.30	0.08	0.31
9	lysoPC a C28:0	0.35	0.17	0.30	0.47	0.1	0.48	0.30	0.09	0.27
10	lysoPC a C28:1	0.47	0.18	0.45	0.67	0.21	0.62	0.50	0.13	0.456
11	PC aa C34:4	1.62	0.67	1.62	1.85	0.68	1.85	1.08	0.31	0.97
12	PC aa C38:1	1.19	0.51	1.20	0.99	0.38	0.94	0.53	0.29	0.63
13	PC ae C36:4	18.87	5.01	19.10	16.27	3.25	15.60	11.45	3.81	10.34
14	PC ae C38:4	14.38	3.23	14.05	13.75	2.41	13.20	10.91	2.52	10.70
15	PC ae C38:5	17.87	4.07	17.80	16.29	2.37	17.00	12.46	3.13	11.75
16	PC ae C40:1	1.31	0.30	1.31	1.33	0.27	1.25	1.03	0.18	1.06
17	PC ae C42:1	0.31	0.088	0.31	0.37	0.06	0.37	0.29	0.07	0.28
18	PC ae C42:4	0.68	0.27	0.77	0.65	0.25	0.67	0.39	0.22	0.39

PD—Parkinson’s disease; APDs—atypical parkinsonian disorders; DMA—dimethylarginine; C18:2—octadecadienylcarnitine; PC aa/ae C *X*:Y—diacyl(aa)/acyl-alkyl (ae) phosphatidylcholine with the total number of carbon atoms denoted by *X* and the number of double bonds indicated by *Y*.

**Table 2:** The results of the post-hoc analyses of serum metabolites.

Serum				
No	Metabolite	Post Hoc Test *p*-Values		
		C vs. PD	C vs. APDs	PD vs. APDs
1	C18:2 ^a^	0.049849	*NS*	*NS*
2	Ornithine ^a^	0.045345	*NS*	*NS*
3	Tyrosine ^b^	0.015431	*NS*	*NS*
4	Putrescine ^b^	0.004395	0.011878	*NS*
5	Spermidine ^a^	0.013458	0.000915	*NS*
6	t4-OH-Proline ^b^	0.006106	0.00141	*NS*
7	Total DMA ^a^	*NS*	0.001545	*NS*
8	lysoPC a C28:0 ^b^	*NS*	*NS*	0.041845
9	lysoPC a C28:1 ^b^	0.038853	*NS*	*NS*
10	PC aa C34:4 ^a^	*NS*	*NS*	0.042019
11	PC aa C38:1 ^a^	*NS*	0.008727	*NS*
12	PC ae C36:4 ^a^	*NS*	0.00344	*NS*
13	PC ae C38:4 ^a^	*NS*	0.046699	*NS*
14	PC ae C38:5 ^a^	*NS*	0.007192	*NS*

^a^ ANOVA; ^b^ Kruskal–Wallis; *NS—*not significant; C—control; PD—Parkinson’s disease; APDs—atypical parkinsonian disorders; DMA—dimethylarginine; C18:2—octadecadienylcarnitine; PC aa/ae C *X*:*Y*—diacyl(aa)/acyl-alkyl (ae) phosphatidylcholine with the total number of carbon atoms denoted by *X* and the number of double bonds indicated by *Y*.

**Table 3:** A list of the metabolites determined in the cerebrospinal fluid of patients and identified as significantly different between the analyzed groups based on either analysis of variance (ANOVA) or non-parametric Kruskal–Wallis ANOVA, depending on the normality of distribution and variance equality. All values are expressed in micromoles.

CSF										
No.	Metabolite	Control (*n* = 10)			PD (*n* = 11)			APDs (*n* = 8)		
		Mean	SD	Median	Mean	SD	Median	Mean	SD	Median
1	Tyrosine	8.93	3.09	7.98	14.09	4.44	13.00	13.42	5.81	10.65
2	Putrescine	0.13	0.03	0.14	0.19	0.05	0.20	0.16	0.05	0.16
3	t4-OH-Proline	0.42	0.13	0.39	1.06	0.88	0.76	0.74	0.15	0.74
4	total DMA	0.15	0.07	0.13	0.18	0.06	0.19	0.25	0.05	0.24

PD—Parkinson’s disease; APDs—atypical parkinsonian disorders; t4-OH-Pro—trans-4-hydroxyproline; DMA—dimethylarginine.

**Table 4:** The results of the post-hoc analyses of the cerebrospinal fluid metabolites.

CSF				
No	Metabolite	Post-Hoc Test *p*-Values		
		C vs. PD	C vs. APDs	PD vs. APDs
1	Tyrosine ^b^	0.008478	*NS*	*NS*
2	Putrescine ^a^	0.01245	*NS*	*NS*
3	t4-OH-Proline ^b^	0.000673	0.00936	*NS*
4	total DMA ^a^	NS	0.011032	*NS*

^a^ ANOVA; ^b^ Kruskal–Wallis; *NS—*not significant; C—control group; PD—Parkinson’s disease; APDs—atypical parkinsonian disorders; t4-OH-Pro—trans-4-hydroxyproline; DMA—dimethylarginine.

**Table 5:** Demographic and clinical features of the patients with PD, atypical parkinsonian disorders (APDs) patients, and the controls.

Feature	PD (*n* = 10), Mean ± SD	Controls (*n* = 10), Mean ± SD	APDs (*n* = 8), Mean ± SD
Male/female (*N*)	4/6	7/3	3/5
Age (years)	60 ± 52	52 ± 13	63 ± 12
Disease duration (years)	7 ± 5	-	7 ± 7.5
LEDD	804 ± 514	-	641 ± 446
HY	2 ± 1	-	
UPDRS III	19 ± 12	-	
Dementia	0	0	1
Depression	0	0	3
BMI	27 ± 5	24 ± 3	26 ± 4
Other drugs	Levothyroxine, aspirin, quetiapine, and angiotensin-converting enzyme inhibitor	Levothyroxine, beta-blockers, statins, and angiotensin-converting enzyme inhibitor	Quetiapine, sertraline, beta- blockers, statins, angiotensin- converting enzyme inhibitor, and tiapride

LEDD—L-dopa equivalent daily dose; HY—modified Hoehn and Yahr rating scale; UPDRS III—Unified Parkinson’s Disease Rating Scale Part III.

## Data Availability

Data is contained within the article.
